# Management of Submassive Bilateral Pulmonary Embolism in an Adolescent Female

**DOI:** 10.1155/2021/1678528

**Published:** 2021-09-29

**Authors:** Adeline Yi Ling Lim, John Roy, Ajay Kevat

**Affiliations:** ^1^Department of Respiratory and Sleep Medicine, Queensland Children's Hospital, 501 Stanley Street, South Brisbane, Queensland 4101, Australia; ^2^Department of Haematology, Queensland Children's Hospital, 501 Stanley Street, South Brisbane, Queensland 4101, Australia

## Abstract

Pulmonary embolism (PE) is a rare presentation in the pediatric population. We report a case of submassive PE in an adolescent female following commencement of a combined oral contraceptive pill (COCP). In the setting of cardiac dysfunction, she received systemic thrombolysis with significant reduction of clot burden and clinical improvement objectively demonstrated shortly thereafter. This case highlights challenges in clinical decision-making regarding surgical or catheter-based interventions versus medical management approaches when addressing life-threatening PE in children. Our case demonstrates that submassive PE in pediatrics can be managed successfully with systemic thrombolysis and therapeutic anticoagulation.

## 1. Introduction

Pulmonary embolism (PE) is a rare presentation in the pediatric population, with an estimated incidence of 8.6–57 in 100,000 in hospitalized children and approximately 0.14–0.9 in 100,000 in all children in the community [1]. Risk factors for PE in children include malignancy, congenital heart disease, systemic lupus erythematosus, renal disease, trauma, and acquired and inherited thrombophilia [2]. Submassive and massive pulmonary embolism have previously been reported in the adolescent population, with a majority having predisposing comorbidities [3].

Hormonal contraception can provoke venous thromboembolism (VTE), where the estrogen component of this treatment is known to cause resistance to activated protein C and increased thrombosis risk [4]. In a large cohort study of females aged 15 to 49 years using the combined oral contraceptive pill (COCP), approximately 19.5% were aged between 15 and 19 years, with 173 pulmonary embolisms reported (17.5 per 100,000 women years) in this age group. The study also revealed a lower risk of PE with smaller estrogen doses in combination with levonorgestrel, as compared to desogestrel or gestodene [5]. A recent systematic review did not reveal a significant risk for PE with progestin-only contraceptives [6].

Here, we report the case of an adolescent female who developed provoked submassive bilateral PE with haemodynamic strain post commencement of a COCP. In the absence of clear pediatric-specific evidence, we explored possible management pathways extrapolated from adult and pediatric research and guidelines, with an eventual successful treatment outcome.

## 2. Case Report

A 14-year-old female presented to our pediatric tertiary hospital emergency department (ED) with acute dyspnea and pleuritic chest pain following a presyncopal episode while walking up a flight of stairs. She was persistently tachycardic with a heart rate of 120 beats per minute at rest and higher with minimal exertion, tachypneic (20 breaths per minute), normotensive (mean arterial pressure 80-88 mmHg) and had mild hypoxia with baseline saturations of approximately 90% at rest, decreasing to the mid-80s with mobilisation. She was commenced on supplemental low flow nasal oxygen at a flow rate of three litres per minute which improved her oxygen saturations.

The patient was otherwise well with no history of recent viral illness, surgery, trauma, or periods of immobilisation. She had however commenced a COCP consisting of ethinyloestradiol (20 micrograms) and levonorgestrel (100 micrograms) ten weeks prior. The COCP was prescribed by her local doctor for a sharp pelvic pain which started with menstruation and persisted for three weeks, with complete resolution while taking the COCP. The patient reached menarche at twelve years old and experienced dysmenorrhea for the first three days of menstruation with menorrhagia. Apart from her maternal grandmother having endometriosis, there was no significant family history including thrombophilia or thromboembolic events. The patient denied smoking or recreational drug use. She was a competitive sports player who, within a month of commencing the COCP, experienced dyspnea and intermittent chest pain on exertion. Although these symptoms initially resolved with rest, her time to recovery gradually increased.

**Figure 1:**
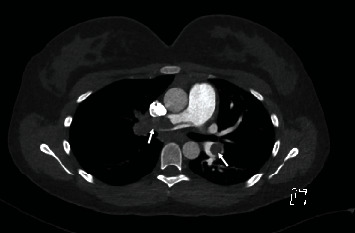
CTPA prior to thrombolysis with pulmonary embolism present (arrows).

In the ED, a chest X-ray (CXR) revealed mild prominence of the pulmonary arteries bilaterally. Blood tests revealed elevated levels of D-dimer, 5.01 mg/L (normal range 0.02–0.49) and cardiac troponin I (cTnI) of 194 Hng/L (normal < 10). Given the history and clinical findings, a computer tomography pulmonary angiogram (CTPA) was performed and confirmed the presence of a saddle PE (Figure 1) with thrombus occluding the right main pulmonary artery, left upper lobe segmental artery, and left interlobar artery. There was also evidence of right heart strain on the CTPA. An urgent echocardiogram revealed systolic dysfunction of the right ventricle (RV) and basal segment dyskinesia, and blood flow through the right pulmonary artery appeared absent. Given the presence of right heart strain, elevated troponin levels, and persistent tachycardia and hypoxia, urgent opinions were sought from multiple pediatric specialists including a respiratory physician, interventional cardiologist, haematologist, and cardiothoracic surgeon.

The patient was administered a 10 milligram (mg) bolus of intravenous alteplase, a recombinant tissue plasminogen activator (tPA), and admitted to the pediatric intensive care unit (PICU) where she then received an infusion of tPA (0.3 mg/kg/h) for 3 hours. The total dose of tPA administered was 80 mg. Following this, the patient was commenced on an unfractionated heparin (UHF) infusion, targeting an anti-Xa level of 0.3–0.7 U/mL. COCP administration was ceased.

Following thrombolysis, the patient's tachycardia resolved, although she remained tachypneic. A repeat echocardiogram within twelve hours showed improvement in the RV systolic dysfunction and definite blood movement through the right pulmonary artery, with ongoing presence of a significant filling defect occupying the vessel. UHF infusion was continued for 48 hours, after which time anticoagulation was changed to 80 mg subcutaneous enoxaparin, a low molecular weight heparin (LMWH), administered twice a day. Given the significant embolic material seen on the initial CTPA, further discussions ensued regarding the potential benefits and risks of catheter-directed intervention with the aim of decreasing clot burden and potentially reducing the risk of chronic thromboembolic pulmonary hypertension (CTEPH).

**Figure 2:**
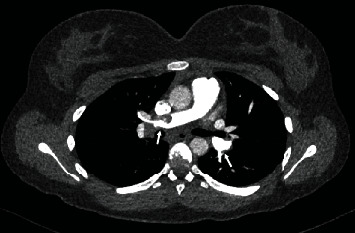
CTPA performed four days post thrombolysis with reduction of pulmonary embolism (arrows).

A repeat CTPA was performed on the fourth day of admission which revealed a substantial decrease in clot burden, no visible saddle embolus, and reduced right heart strain (Figure 2). The patient no longer required supplemental oxygen and made a steady recovery. Eight days post admission, she was transitioned to a direct oral anticoagulant (DOAC), rivaroxaban, at standard adult dosing (15 mg twice a day for three weeks followed by 20 mg daily), prior to discharge home.

**Figure 3:**
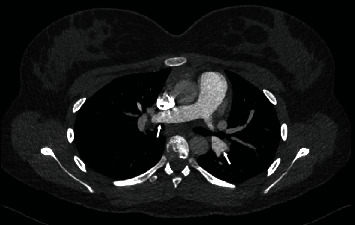
CTPA performed six months post thrombolysis with resolution of pulmonary embolism (arrows).

A thrombophilia screen performed was unremarkable (Table 1). She was referred to an adolescent gynaecologist to explore suitable alternatives to taking the COCP. Outpatient spirometry, body plethysmography, and diffusing capacity tests performed a month post discharge were unremarkable. She returned to her baseline function with resolution of the shortness of breath on exertion. A CTPA and echocardiogram performed six months post presentation revealed complete resolution of all pathological changes, and rivaroxaban therapy was stopped (Figure 3). Written consent was obtained from the patient's parent for this case report.

## 3. Discussion

Pediatric PE often appears clinically silent, although children with PE can present with the classic symptoms including dyspnea, tachypnea, pleuritic chest pain, haemoptysis, cough, and/or syncope [1]. The Wells' criteria and pulmonary embolism rule-out criteria (PERC), while validated as diagnostic prediction tools for PE in adults, have not been shown to be suitable for pediatric use [1]. The D-dimer is a sensitive but not specific test for PE and is most commonly used for its negative predictive value in adults [7].

As clinical presentation and nonimaging diagnostic tests lack specificity, imaging modalities have been used to define the presence of PE. While a CXR is commonly performed, normal findings are found in approximately 24% of patients with PE [8]. PE is therefore more clearly defined with a CTPA or lung scintigraphy. CTPA has largely overtaken scintigraphy as the primary imaging technique for diagnosing PE, with a false-negative rate of <3% (false-positive rate of <10%) in pooled pediatric studies [9]. Lung scintigraphy has also been utilized to diagnose PE in children, although the ventilation-perfusion mismatch can also be present in pneumonia, sickle-cell disease, asthma, arterial stenosis, and certain forms of congenital heart disease, such as the “Fontan circulation” [2]. As significant cooperation is required, younger children may not be able to perform the ventilation component of the scan. CTPA imaging was undertaken in our case as it was most accessible at the time of presentation.

With echocardiography, the presence of RV dysfunction correlates with a higher chance of death, haemodynamic collapse, and/or PE recurrence [10]. Due to the absence of hypotension but presence of RV dysfunction plus elevated cardiac biomarkers and tachycardia, our patient met criteria for an intermediate-high-risk classification according to international guidelines [11]. This classification predicts an 18% (95% CI 9%-30%) risk of a complicated course (defined as 30-day mortality, haemodynamic collapse, or PE recurrence) based on adult data [10]. Our patient was thus managed in the PICU until substantial clinical and echocardiographic improvement was achieved.

Management options for submassive PE with cardiac dysfunction vary from surgical embolectomy to catheter-directed therapy or medical management with systemic thrombolysis and/or anticoagulation [11]. Controversies exist in choosing between these options in adult patients, and there is little available evidence to guide management in children. In our case, urgent surgical embolectomy was not selected due to the significant risk of intraoperative death [12]. We note a previous case report where surgical embolectomy was successfully performed in an adolescent for saddle PE without haemodynamic instability [13].

We considered catheter-based interventions to disrupt and dissolve clot, including catheter-delivered thrombolysis (CDT). A pediatric case series of patients with severe PE managed with CDT (in combination with ultrasound agitation in five procedures) achieved partial or complete resolution of PE without mortality or complications [14]. Complications of CDT for PE include major bleeding such as haemorrhagic stroke in 2-14% and procedural failure in 3-29%, with greater numbers in higher-risk PE [15]. Serious haemorrhagic complications occurred at rates similar to those where systemic thrombolysis was used [16].

Systemic thrombolysis leads to better improvements in pulmonary obstruction, pulmonary artery pressure, and pulmonary vascular resistance in patients with massive or submassive PE, compared with UFH alone [17]. Although systemic thrombolysis was associated with an increased risk of severe bleeding in an adult study [18], a separate review of thrombolysis in pediatric patients for all indications described intracranial haemorrhage as an adverse event occurring predominantly in infancy [19]. The impact of early thrombolysis for acute PE on the risk of developing CTEPH remains unclear [20].

We proceeded with systemic thrombolysis in our patient, with decreased clot burden within four days and complete dissipation after six months. The American Society of Hematology 2018 guidelines recommend anticoagulation for three months or less, or until resolution of the precipitating risk factor in provoked PE, and treatment for six to twelve months for unprovoked PE [21]. Newer DOACs are more appealing for pediatric use given the elimination of injections. Rivaroxaban, a direct inhibitor of factor Xa, was found in a large pediatric randomised controlled trial of VTE treatment to have an equivalent reduction in recurrence risk and thrombotic burden, without increased bleeding when compared with standard anticoagulants [22]. Our patient received oral rivaroxaban for six months without any significant complications.

## 4. Conclusion

PE is a potentially life-threatening but rare condition in children. Our adolescent patient suffered a submassive bilateral PE with cardiac dysfunction after commencing COCP use. While there is no clear consensus on the management of this presentation, we drew upon evidence from published research to inform our treatment. Systemic thrombolysis followed by anticoagulation resulted in complete radiological resolution of thrombus, and the patient made a full recovery without complications. Further studies involving children and adolescents are needed to ascertain the optimal management of PE in these patient subgroups.

## Figures and Tables

**Table 1:** Thrombophilia screen.

Investigations	Results	Reference range
Lupus anticoagulant	Negative	
Antithrombin (U/mL)	0.89	0.83–1.28
Protein C (U/mL)	0.85	0.7–1.3
Protein S (U/mL)	0.82	0.55–1.24
Factor V Leiden	Absent	
Anti-cardiolipin IgG antibodies (CU)	8	<20
Anti-beta 2 glycoprotein I (G units)	0	<20

## Data Availability

All relevant data have been included in the article.
